# Late-phase impact of CMV/EBV reactivation on survival after hematopoietic stem-cell transplantation: a 5-year single-center cohort study

**DOI:** 10.3389/fcimb.2026.1782595

**Published:** 2026-03-25

**Authors:** Shanshan Li, Mei Jia, Liyan Cui

**Affiliations:** 1 Department of Laboratory Medicine, Peking University Third Hospital, Beijing, China; 2 Core Unit of National Clinical Research Center for Laboratory Medicine, Peking University Third Hospital, Beijing, China; 3 Department of Clinical Laboratory, Peking University People’s Hospital, Beijing, China

**Keywords:** CMV, EBV, HSCT, long-term survival, reactivation

## Abstract

**Background:**

Whether simultaneous cytomegalovirus (CMV) and Epstein–Barr virus (EBV) reactivation confers additional late mortality beyond isolated CMV remains controversial.

**Methods:**

We retrospectively analyzed 202 consecutive first-ever hematopoietic stem cell transplantations performed between 2018 and 2019, using twice-weekly PCR surveillance and uniform preemptive therapy. Cox models were used to estimate hazard ratios (HRs) for overall survival (OS) and leukemia-free survival (LFS) at the 1-, 3-, and 5-year landmarks.

**Results:**

Although 1-year outcomes were similar, divergence emerged thereafter: 5-year OS was 19.4% with coreactivation, 36.6% with isolated CMV, and 25.1% with no reactivation (*p* = 0.041); corresponding LFS was 19.4%, 36.6%, and 25.2% (*p* = 0.060). Multivariate analysis identified sustained CMV replication as the dominant late risk factor (OS HR 5.295, 95% CI 1.5888–17.6464; *p* = 0.0067), whereas coreactivation lost significance because EBV clearance shortened the overall viral window. Interestingly, viral enteritis was identified as an independent adverse predictor of 1-year LFS (HR 6.2453, 95% CI 1.3245–7.2376; *p* = 0.0010).

**Conclusions:**

Late mortality is driven by persistent CMV-driven endothelial injury rather than transient EBV coreactivation. Extending PCR surveillance from day 100 to year 2 and targeting chronic low-level CMV should be prioritized to improve long-term transplantation success.

## 1 Introduction

Allogeneic hematopoietic stem cell transplantation (allo-HSCT) remains the most potent curative option for an expanding spectrum of malignant and non-malignant hematologic disorders. However, its success is continuously threatened by procedure-related morbidity and late mortality (Hou et al., 2025). Overall survival (OS) and leukemia-free survival (LFS) are therefore accepted as cardinal oncologic endpoints that capture both relapse-free and transplant-related mortality. However, they are heavily modulated by infectious complications that arise while donor-derived immunity is being rebuilt (Cavallaro et al., 2024; Yang et al., 2025). Among these, the reactivation of viruses - with cytomegalovirus (CMV) and Epstein-Barr virus (EBV) being the most common - poses a threat to patients who are recovering. In 30% to 80% of seropositive recipients, CMV DNAemia can be detected within the first 100 days, while EBV DNAemia is recorded in 20% to 50% of the recipients; the incidence rate varies depending on the donor type, the source of the graft, and the intensity of immunosuppression (Li et al., 2022; Kong et al., 2024). Both viruses are biologically engineered to persist lifelong in latently infected cells of either host or donor origin, and the profound lymphopenia that follows conditioning creates an ecological niche for their recrudescence once protective antigen-specific immunity falls below a critical threshold (Pociupany et al., 2025; Sun et al., 2025). CMV reactivation is no longer regarded as an isolated virological phenomenon, but rather as a sentinel marker of systemic immune dysfunction. It significantly increases the risk of secondary bacterial and fungal superinfections and serves as an independent predictor of non-relapse mortality. This adverse outcome stems from both direct end-organ damage—manifesting as pneumonitis, gastroenteritis, and retinitis—and indirect immunomodulatory effects that exacerbate graft-versus-host disease (GVHD) while compromising graft-versus-leukemia (GVL) activity (Ljungman et al., 2025; Zhu et al., 2025). Similarly, EBV reactivation culminates in a clinical spectrum ranging from asymptomatic viremia to fulminant post-transplant lymphoproliferative disorder (PTLD), a potentially fatal proliferation of transformed B cells driven by the viral oncoprotein LMP-1 when cytotoxic T-cell surveillance is impaired (Todorovic et al., 2024; Jiang et al., 2025). Because complications resulting from viral infection can affect organ transplant outcomes, either through direct viral toxicity or immune system complications, it is important to understand the impact of CMV and EBV on OS and LFS over 1, 3, and 5 years.

## 2 Materials and methods

### 2.1 Study cohort

**Figure 1:**
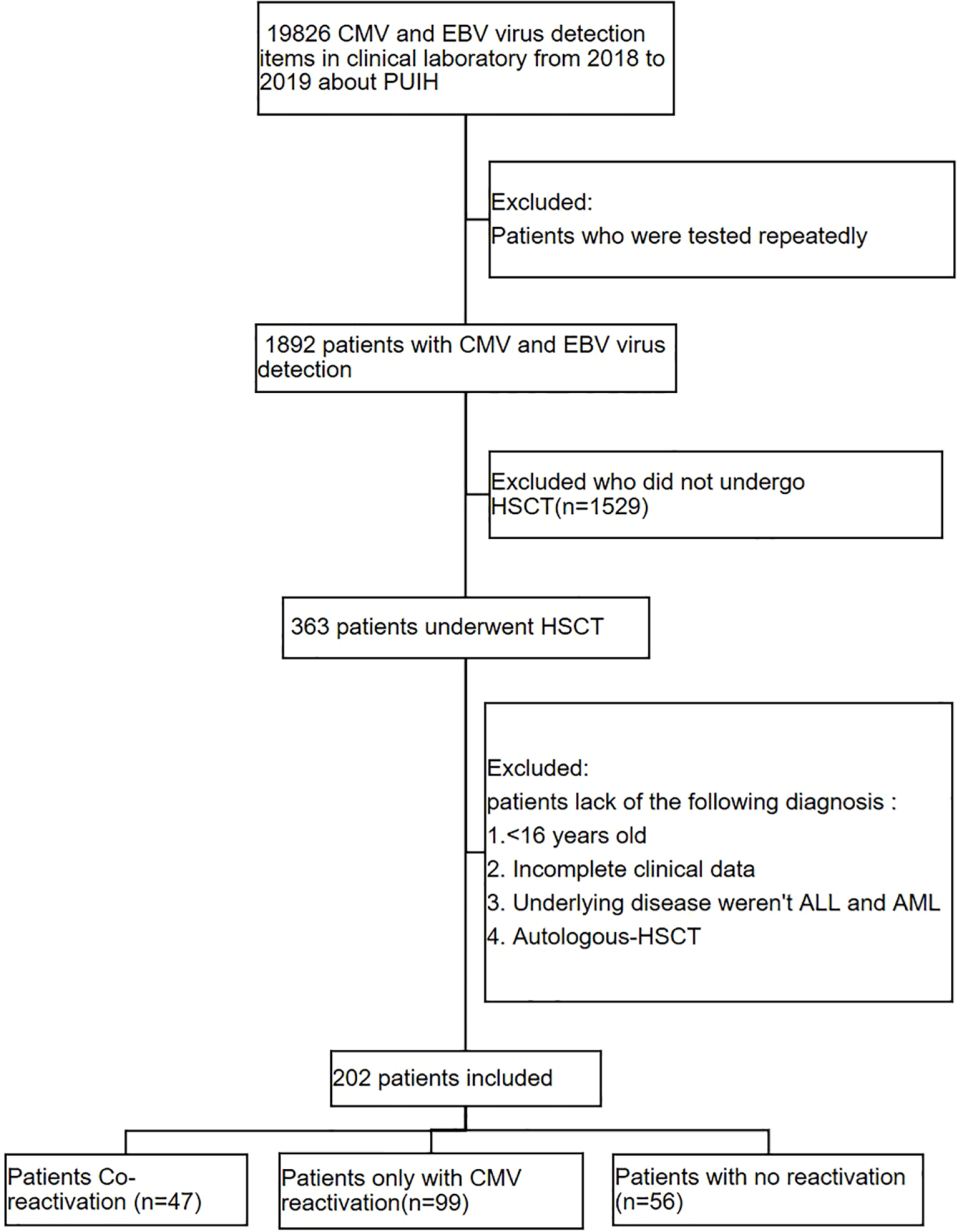
Patient inclusion flowchart. From 18,826 CMV/EBV tests performed in the clinical laboratory of Peking University International Hospital (PUIH) during 2018–2019, 1,892 unique patients with viral detection results were identified. After excluding 1,529 who did not undergo hematopoietic stem-cell transplantation (HSCT), 363 HSCT recipients remained. Application of additional exclusion criteria (age <16 years, incomplete clinical data, underlying disease other than ALL or AML, EBV-only reactivation) yielded the final analytic cohort of 202 patients, comprising 47 with CMV/EBV co-reactivation, 99 with CMV-only reactivation, and 56 without viral reactivation.

This retrospective study enrolled 202 consecutive patients who underwent their first allogeneic hematopoietic stem cell transplantation (allo-HSCT) at the Institute of Hematology, Peking University People’s Hospital, between January 2018 and January 2019 (**Figure 1**). Medical charts were systematically reviewed to extract relevant data. This study was exempt from ethical approval by the Peking University People’s Hospital Ethics Committee because of its retrospective nature and the use of anonymized and de-identified data. The study protocol complied with the ethical principles of the Declaration of Helsinki.

### 2.2 Transplantation protocols

Conditioning regimens were tailored according to donor type. Patients undergoing unrelated donor (URD) or haploidentical (haplo) HSCT received a modified rabbit antithymocyte globulin-based regimen (2.5 mg/kg/day; Sang Stat, Lyon, France) combined with busulfan/cyclophosphamide (BU/CY; busulfan 9.6 mg/kg intravenously divided over days −8 to −6, cyclophosphamide 1.8 g/m²/day on days −5 to −4). Those with matched sibling donors (MSD) were conditioned using the standard BU/CY protocol. G-CSF–mobilized peripheral blood stem cells (PBSCs) were infused in MSD and haploidentical HSCT recipients, whereas URD recipients received unmanipulated PBSCs (3.0 × 10^8^ cells/kg) following G-CSF priming (5 µg/kg daily for 5–6 days) (Wang et al., 2014; Wang et al., 2015; Xu et al., 2018). GVHD prophylaxis consisted of mycophenolate mofetil, cyclosporine, and short-course methotrexate in all cases.

### 2.3 Viral surveillance and management

Reactivation of CMV and EBV was assessed using plasma samples via real-time quantitative polymerase chain reaction (PCR). CMV and EBV surveillance following allo-HSCT followed a risk-stratified protocol: weekly quantitative PCR monitoring from engraftment through day 100 for all patients, with high-risk recipients (D-/R+, cord blood, PTCy-based haploidentical HSCT, or active GVHD) continuing weekly testing until immunosuppression withdrawal. Beyond day 100, standard-risk patients were transitioned to biweekly or monthly surveillance, whereas high-risk individuals were monitored intensively for up to 6–12 months post-transplant, guided by CMV-specific cellular immune reconstitution assays when available. All patients were administered ganciclovir between days −9 and −2. Preemptive therapy with either intravenous foscarnet (90 mg/kg/day) or intravenous ganciclovir (5 mg/kg, twice daily) was initiated upon confirmed CMV viremia reactivation. Therapy was continued until CMV DNA was undetectable in two consecutive tests. Antiviral agents such as foscarnet were administered to patients with EBV reactivation. Moreover, rituximab was administered when EBV viremia persisted or progressed to EBV disease. Salvage therapy involved EBV-specific cytotoxic T lymphocytes (CTLs) (Huang et al., 2009; Liu et al., 2010).

### 2.4 Definitions

Myeloid engraftment was defined as the first of three consecutive days with an absolute neutrophil count ≥0.5 × 10^9^/L. Platelet engraftment was recorded as the first of seven consecutive days with a platelet count ≥20 × 10^9^/L without transfusion support. Viral pneumonia encompassed lower respiratory tract infections attributable to influenza A/B, paramyxoviruses, CMV, EBV, respiratory syncytial virus, adenovirus, and rhinoviruses. CMV reactivation was declared when plasma DNA reached ≥1,000 copies/mL on two successive tests; the corresponding cutoff for EBV was ≥500 copies/mL. Concurrent CMV and EBV reactivation was recorded if both thresholds were exceeded within the first year post-HSCT. The duration of viremia was calculated from the initial positive PCR result to the first two consecutive negative results. Time to relapse was measured from transplantation to hematological recurrence. Overall survival (OS) and leukemia-free survival (LFS) were defined as the interval from HSCT to death from any cause or relapse/progression.

### 2.5 Statistical analysis

Categorical variables were compared using the χ² test or Fisher’s exact test, as appropriate. Continuous variables were analyzed using the Mann–Whitney U test. Multivariate Cox regression models were constructed after verification of the proportional hazards assumption and linearity of time-dependent covariates. Variables yielding *p* < 0.10 in univariate screening were entered into the multivariate model; hazard ratios (HRs) and 95% confidence intervals (CIs) were derived. All analyses were performed using SPSS (version 25.0; IBM, Armonk, NY, USA) and GraphPad Prism 8.0.1 (GraphPad Software, Inc.). Statistical significance was defined as a two-tailed *p*-value < 0.05.

## 3 Results

### 3.1 Patient characteristics and clinical outcomes

Among the 202 patients who received their first allogeneic hematopoietic stem cell transplant between January 2018 and January 2019, 70.3% were ≤40 years of age and 57.9% were male (**Table 1**). Acute myeloid leukemia accounted for 60.7% of underlying diseases, whereas acute lymphoblastic leukemia constituted the remaining 39.3%. The vast majority (92.6%) underwent transplantation within 12 months of diagnosis, and 97.6% were in first complete remission (CR1) at the time of conditioning, indicating a standard-risk disease profile. Sibling donor transplants represented the most common donor–recipient constellation (41.6%), followed by parent-to-child (37.1%), children-to-parent (15.3%), cousin (2.0%), and fully matched unrelated donors (4.0%). Consequently, 71.8% of the cohort were haploidentical, 24.2% received HLA-identical sibling grafts, and only 4.0% received 10/10 matched unrelated grafts. Overall, 60.4% of HSCTs were ABO compatible and 39.6% were ABO mismatched; donor–recipient sex concordance was present in 51.0%. Infused graft characteristics revealed a median mononuclear cell dose of 8.95 × 10^8^/kg and a median CD34^+^ cell dose of 3.00 × 10^6^/kg. Of note, patients who later developed CMV/EBV coreactivation received significantly lower MNC counts (8.60 vs 8.95 × 10^8^/kg; *p* = 0.041), whereas CD34^+^ content showed only a nonsignificant trend toward reduction (1.78 vs 2.53 × 10^6^/kg; *p* = 0.116). Age distribution differed across viral groups; individuals <40 years constituted 83.0% of the coreactivation subset but only 55.4% of the nonreactivation group (*p* = 0.007), suggesting that younger recipients, possibly through more robust immune reconstitution, may paradoxically exhibit earlier viral replication kinetics. Haploidentical transplantation was markedly overrepresented among those with subsequent coreactivation (95.7% vs 32.1%; *p* < 0.001), underscoring the heightened viral risk inherent in T-cell-replete haplo platforms.

**Table 1:** Characteristics of the patients.

Characteristics	Total	Co-reactivation group	Only CMV reactivation group	No reactivation group	*P*
No. of patients (%)	202	47(23.3)	99(49.0)	56(27.7)	
Gender no.(%)					0.571
Male,	117(57.9)	26(12.9)	61(30.2)	30(14.8)	
Female	85(42.1)	21(10.4)	38(18.8)	26(12.9)	
Age, median(rang)					0.007
>=40 years	60(29.7)	8(4.0)	27(13.4)	25(12.4)	
<40 years	142(70.3)	39(19.3)	72(35.6)	31(15.3)	
Underlying disease, no.(%)					0.636
AML	123(60.7)	26(12.9)	61(30.2)	36(17.3)	
ALL	79(39.3)	21(10.4)	38(18.8)	20(9.9)	
Donor-recipient relationship, no.(%)					0.000
Mother/Father	75(37.1)	30(14.9)	36(17.8)	9(4.5)	
Son/Daughter	31(15.3)	5(2.5)	19(9.4)	7(3.5)	
Sibling	84(41.6)	11(5.4)	35(17.3)	38(18.7)	
Cousin	4(2.0)	1(0.5)	2(1.0)	1(0.5)	
Unrelated donor	8(4.0)	0(0.0)	7(3.5)	1(0.5)	
HLA match, no.(%)					0.000
Haploidentical	145(71.8)	45(22.2)	82(40.7)	18(8.9)	
Identical	49(24.2)	2(1.0)	10(5.0)	37(18.4)	
Unrelated donor	8(4.0)	0(0.0)	7(3.5)	1(0.5)	
Donor-Recipient gender					0.120
Identical	103(51.0)	25(12.4)	55(27.7)	22(10.9)	
Different	99(49.0)	21(10.4)	44(21.8)	34(16.8)	
ABO match					0.463
matched	122(60.4)	32(15.8)	57(28.3)	33(16.3)	
mismatched	80(39.6)	15(7.4)	42(20.8)	23(11.4)	
Time from diagnosis to transplantation					0.871
Less than 1 year	187(92.6)	44(21.8)	92(45.5)	51(25.2)	
More than 1 year	15(7.4)	3(1.5)	7(3.5)	5(2.5)	
Disease status					0.720
CR1	195(97.6)	45(96.0)	95(97.4)	55(98.8)	
CR2 or NR	7(2.4)	2(4.0)	4(2.6)	1(1.2)	
MNCs in transplant (×10^8^/kg)	8.95(2.69)	8.60(2.65)	8.95(2.89)	7.99(2.39)	0.041
CD34+ cells in transplant (×10^6^/kg)	3.00(1.72)	1.78(1.80)	2.53(1.77)	2.34(1.54)	0.116

Clinical outcome analyses demonstrated that neutrophil engraftment was achieved at a median of 13 days across all groups; however, coreactivated patients showed a marginally faster recovery (13 vs 14 days; *p* = 0.044) (**Table 2**). Platelet engraftment occurred at a median of 15 days, with no intergroup differences. Hemorrhagic cystitis, largely of viral etiology, was significantly more common in patients with CMV or EBV reactivation (26.7% and 13.9% vs 5.0% in the no-reactivation group; *p* < 0.001). In contrast, organ-specific viral syndromes remained rare; viral pneumonitis was recorded in only six episodes (3.0%) and enteritis in 3 cases (1.5%), without significant intergroup differences. While 1-year OS and LFS were comparable across groups (*p* < 0.2), the gap widened progressively thereafter; by 3 years, patients with CMV/EBV coreactivation showed the lowest OS (20.3%) and LFS (19.3%), with the OS difference approaching significance (*p* = 0.051) and LFS already significant (*p* = 0.028). This divergence persisted for 5 years, when coreactivation remained associated with the poorest OS (19.4%; *p* = 0.041) and a trend toward inferior LFS (19.4%; *p* = 0.060), underscoring the sustained adverse impact of dual herpesvirus replication on long-term transplant outcomes.

**Table 2:** The impact of CMV and EBV reactivation on clinical outcomes.

Clinical outcomes	Co-reactivation group	CMV reactivation group	No reactivation group	P value
Neutrophil engraftment				
Planted(days)	13(3)	13(4)	14(4)	0.044
Planted(%)	47(23.3)	98(48.5)	54(26.7)	0.375
Platelet engraftment				
Planted(days)	15(10)	15(8)	14(5)	0.389
planted(%)	16(7.9)	33(16.3)	15(7.4)	0.649
GVHD no.(%)	31(64.0)	69(70.1)	31(62.1)	0.196
a GVHD no.(%)	19(9.4)	39(19.3)	10(5.0)	0.005
Grade I–II	15(7.4)	34(16.8)	9(4.5)	0.889
Grade III–IV	3(1.5)	6(3.0)	1(0.5)	
c GVHD no.(%)	7(3.5)	23(11.4)	20(10.0)	
Hemorrhagic cystitis, no. (%)	28(13.9)	54(26.7)	10(5.0)	0.000
Viral pneumonitis, no. (%)	2(1.0)	4(2.0)	0(0.0)	0.350
Viral enteritis, no. (%)	1(0.5)	2(1.0)	0(0.0)	0.557
Overall survival in 1 year after HSCT no. (%)	44(93.6)	90(90.9)	55(98.2)	0.205
Leukemia free survival in 1 year after HSCT no. (%)	40(85.1)	80(80.8)	46(82.1)	0.818
Overall survival in 3 years after HSCT no. (%)	41(87.2)	76(76.8)	51(91.0)	0.051
Leukemia free survival in 3 years after HSCT no. (%)	39(83.0)	73(73.7)	51(91.0)	0.028
Overall survival in 5 years after HSCT no. (%)	37(78.7)	70(70.7)	48(85.7)	0.041
Leukemia free survival in 5 years after HSCT no. (%)	37(19.4)	68(35.6)	48(25.1)	0.060

Early transplant outcomes and long-term survival of the same cohort, grouped by CMV/EBV reactivation status.

Neutrophil engraftment is defined as the first of 3 consecutive days with ANC ≥0.5×10^9^/L; platelet engraftment as the first of 7 consecutive days with platelet count ≥20×10^9^/L without transfusion. aGVHD and cGVHD are graded according to MAGIC/NIH criteria. Survival probabilities are actuarial estimates at 1, 3 and 5 years after HSCT.

### 3.2 Univariate analysis of CMV or EBV reactivation and GVHD on the outcomes of HSCT patients

**Figure 2:**
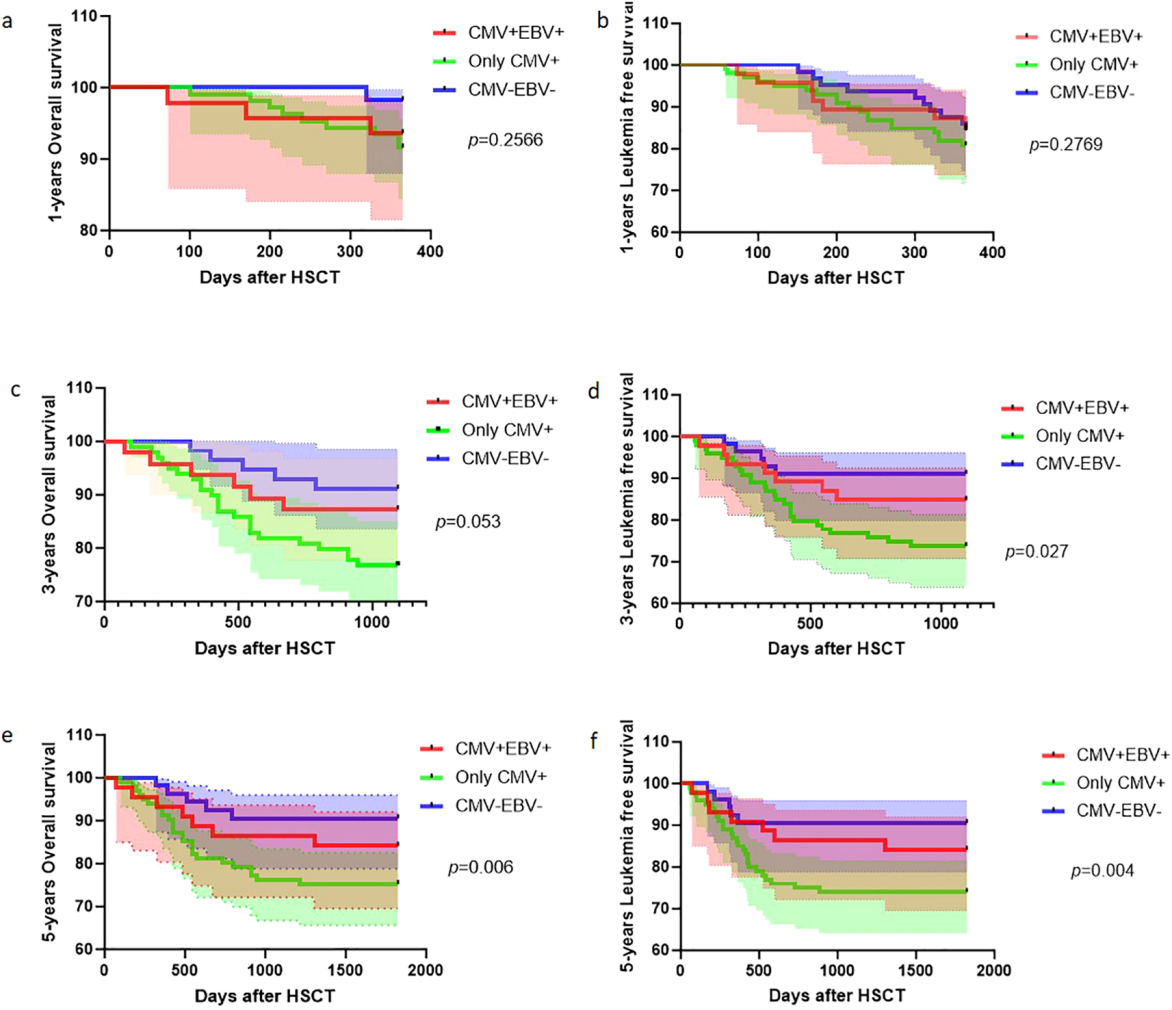
Cumulative incidence of CMV and EBV re-activation after HSCT. **(A–F)** Kaplan–Meier curves stratified by baseline CMV/EBV serostatus: CMV+EBV+ (red), only-CMV+ (blue), CMV–EBV– (black). Time is measured from the day of HSCT. P values (log-rank test) are shown for each panel.

Kaplan–Meier survival analyses comparing CMV/EBV reactivation status after HSCT at 1, 3, and 5 years are presented in six panels. **Figures 2A, B** demonstrate the 1-year OS and LFS probabilities for three cohorts—CMV+EBV+ coreactivation, CMV-only reactivation, and CMV–EBV–no-reactivation controls—with no significant statistical difference. **Figures 2C, D** illustrate 3-year OS, with the CMV+EBV+ cohort showing marginally inferior OS compared with controls and the CMV-only group also demonstrating significantly reduced OS (*p* = 0.053). For 3-year LFS, a statistically significant difference was observed among the three groups (**Figure 2D**, p = 0.027). The CMV+EBV+ cohort exhibited marginally lower LFS than controls, whereas the CMV-only group showed a statistically significant reduction. At the 5-year time point, LFS and OS trends continued from the 3-year analysis, with the CMV-only group demonstrating the most pronounced decline compared with controls. The CMV+EBV+ cohort also exhibited reduced survival outcomes (**Figures 2E, F**; *p* = 0.006; *p* = 0.004).

**Figure 3:**
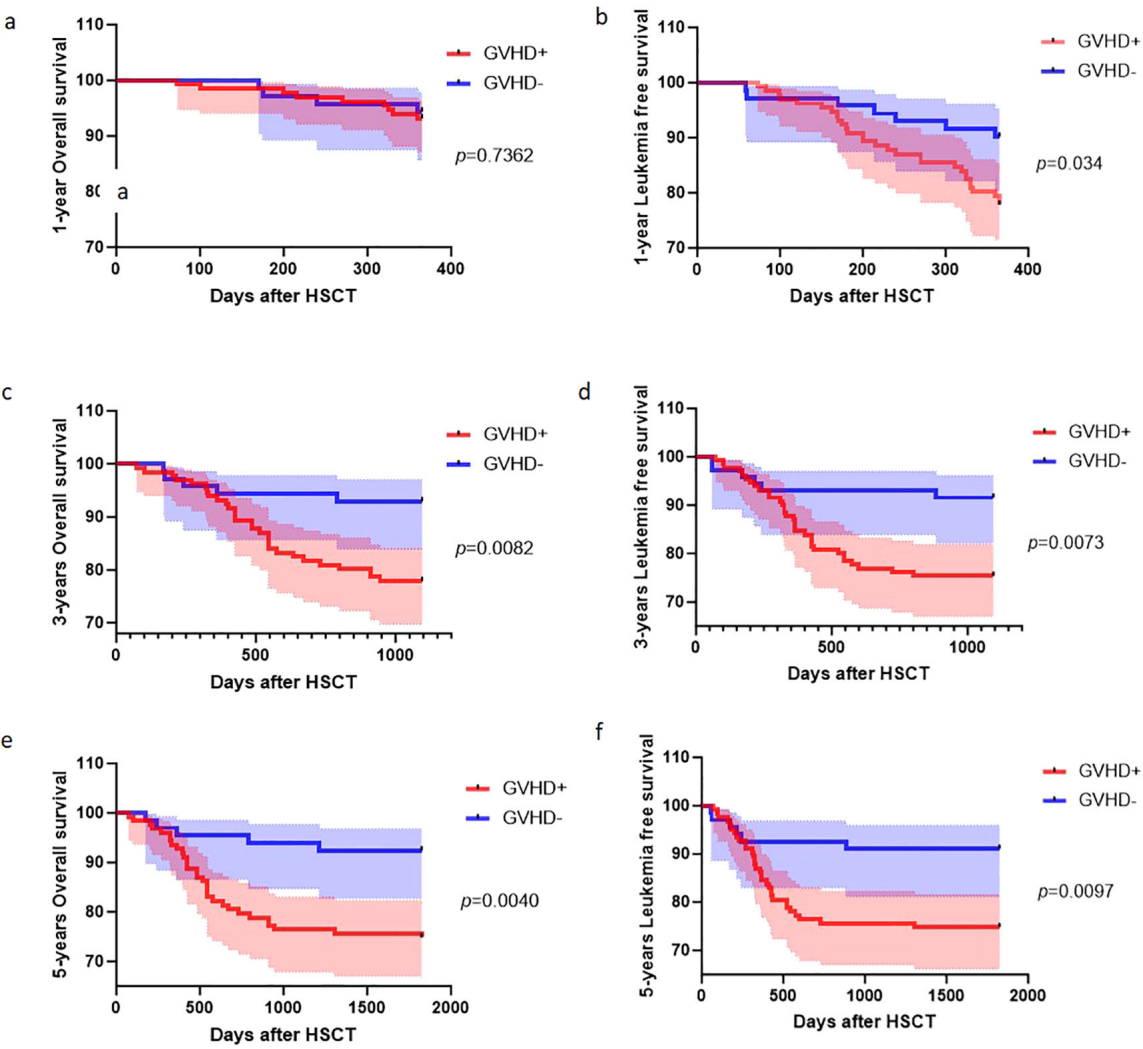
Cumulative incidence for OS and LFS according to acute GVHD status after HSCT. **(A–F)** Kaplan–Meier curves comparing patients who developed acute GVHD (GVHD+, red) with those who did not (GVHD–, blue). Time is measured from the day of HSCT. P values (log-rank test) are shown for each panel.

Long-term survival outcomes stratified by GVHD status are shown in **Figure 3**. Kaplan–Meier analysis revealed significant divergence in both LFS and OS between GVHD+ and GVHD– cohorts over extended follow-up. For LFS, the GVHD+ group demonstrated a statistically significant decrease compared with the GVHD– cohort at all evaluated time points (1, 3, and 5 years), with the between-group disparity progressively increasing over time. No statistically significant difference was observed between the GVHD+ and GVHD– groups at the 1-year follow-up. However, the survival curves began to separate thereafter, with the GVHD+ cohort demonstrating significantly reduced OS at both the 3- and 5-year follow-ups. This delayed divergence mirrors the trajectory observed for OS, wherein the survival disadvantage in the GVHD+ population became more pronounced with longer follow-up duration.

### 3.3 Risk factors for overall survival and leukemia-free survival

**Figure 4:**
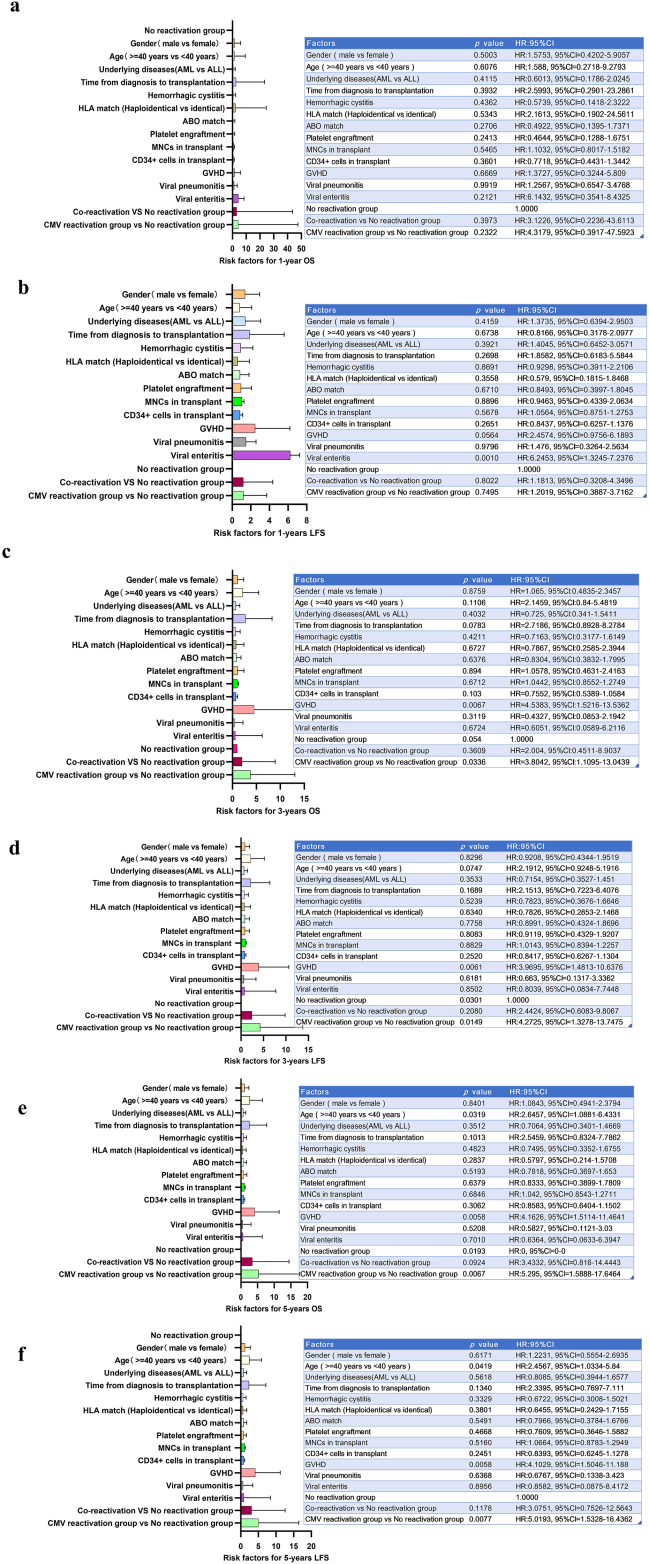
Forest plots of risk factors for overall survival (OS) and leukemia-free survival (LFS) after HSCT. **(A–F)**, Hazard ratios (HR) with 95% confidence intervals for 1-year OS, 1-year LFS, 3-year LFS, 3-year OS, 5-year OS and 5-year LFS are shown. Variables included: underlying disease, time from diagnosis to transplantation, HLA match, ABO match, hemorrhagic cystitis, platelet engraftment, ANCs, MNCs and CD34+ cell counts in the graft, acute GVHD, viral enteritis and viral pneumonia.

**Figure 4** distills the independent determinants of post-transplant OS and LFS into a coherent hierarchy of hazard ratios across three clinically relevant landmarks—1, 3, and 5 years after allo-HSCT.

The forest plot summarizes univariate hazard ratios for 1-year OS and LFS after allo-HSCT (**Figures 4A, B**). Across both endpoints, classic baseline variables—sex, age ≥40 years, underlying disease (AML vs ALL), time from diagnosis to transplantation, ABO and HLA matching, and stem cell dose (MNCs and CD34^+^)—yielded HRs close to 1.0, with 95% confidence intervals spanning unity, indicating no significant impact on early mortality or relapse. In contrast, viral enteritis emerged as the dominant risk factor, conferring a sixfold increase in the risk of LFS failure (HR 6.2453, 95% CI 1.3245–7.2376; *p* = 0.010). GVHD showed a strong trend toward adverse LFS (HR 2.46, *p* = 0.056), whereas coreactivation and isolated CMV reactivation carried HRs of approximately 1.2 relative to the no-reactivation group, but neither reached statistical significance (*p* ≥ 0.7).

When the analytical horizon was extended to 3-year OS and LFS, the prognostic landscape shifted toward viral and immunological variables. CMV reactivation, aggregated as a time-dependent covariate, achieved independent significance for OS (HR 3.8042, 95% CI 1.1095–13.0435; *p* = 0.0336). Concurrently, GVHD achieved independent significance for OS (HR 4.5383, 95% CI 1.5216–13.5298; *p* = 0.0067). The 3-year LFS forest plot recapitulates this hierarchy. GVHD emerged as a colinear driver, conferring a 3.9695-fold increased risk of relapse or death (95% CI 0.1317–3.0362; *p* = 0.0061), while CMV reactivation also achieved independent significance for LFS (HR 4.2725, 95% CI 1.3278–13.3726; *p* = 0.0149).

By the 5-year landmark, the cumulative attrition attributable to late viral reactivation had fully manifested. CMV reactivation emerged as the strongest independent risk factor, conferring a significant increase in the risk of death (HR 5.295, 95% CI 1.5888–17.6464; *p* = 0.0067) and relapse (HR 6.0193, 95% CI 1.5328–16.4362; *p* = 0.0077), even after adjustment for age, disease status, donor type, and prior GVHD. The absence of hemorrhagic cystitis in the late-phase model suggests that its impact is front-loaded. **Figure 4** illustrates the central finding of our study: the graded escalation of coreactivation from non-significant (1 year) to dominant (5 years) underscores the need to extend virologic surveillance beyond the conventional monitoring period.

## 4 Discussion

The present study identifies CMV and EBV reactivation as time-dependent, independent determinants of late mortality and relapse after modern T-cell-replete HSCT. The 5-year OS and LFS curves separated early, within the first 100 days, but continued to diverge over time, suggesting that each episode of detectable viremia may have a sustained impact on immune surveillance. Our findings complement mechanistic studies showing that CMV IE-1 (Stranavova et al., 2019) and EBV LMP-1 (Bollard et al., 2014; Cho et al., 2015) proteins upregulate PD-L1 on professional antigen-presenting cells, thereby creating a “cold” tumor microenvironment that favors leukemic escape. The fact that reactivation had a stronger association with outcomes than the disease risk index, stem cell dose, or chronic GVHD in the final multivariate model supports the concept that successful transplant outcomes depend on effective viral management in addition to tumor biology and conditioning intensity (Gerbitz et al., 2023; Barkhordar et al., 2025). Consequently, future risk-scoring algorithms should incorporate longitudinal viral load metrics alongside traditional variables, and clinical trials testing late-phase antiviral prophylaxis or adoptive T-cell therapy should be stratified according to early dual viremia status.

Several findings challenge the prevailing assumptions and warrant further consideration. First, the higher infection rate observed in younger patients contradicts the assumption that immunosenescence is the primary driver of herpesvirus replication; instead, accelerated thymic output in the <40-year cohort may generate a larger pool of naïve cells that serve as early targets for CMV and EBV, potentially facilitating viral replication until memory inflation is established (Douek et al., 2000; Marty et al., 2017). Second, the stepwise escalation of hazard ratios, from non-significant at 1 year to dominant at 5 years, suggests that the current 100-day surveillance window may be insufficient; late-onset CMV disease and EBV-PTLD continue to occur beyond the second year, supporting extended monitoring in high-risk seropositive recipients. Third, the dissociation between hemorrhagic cystitis and late survival suggests that endothelial injury may act as an early contributor to mortality but may eventually be superseded by immune exhaustion pathways, which should inform the design of composite endpoints that distinguish between early toxicity and late relapse.

Sustained CMV-only replication emerged as an independent predictor of 3- and 5-year mortality, whereas combined CMV/EBV coreactivation lost statistical significance in the late survival models. Biologically, CMV and EBV occupy distinct cellular niches and use different immune evasion strategies that may influence their long-term effects after allo-HSCT. CMV primarily reactivates myeloid-lineage cells and endothelial progenitors, establishing a low-grade vasculitis that may progress over several months. Its IE-1 and US2–11 proteins downregulate HLA class I and secrete viral interleukin-10 (Forker et al., 2019; Stranavova et al., 2019), creating a sustained proinflammatory environment that damages the vascular endothelium of multiple organs (lung, gut, and retina) and exacerbates steroid-refractory GVHD (Hu et al., 2022; Hu et al., 2024). Because endothelial injury is cumulative and only partially reversible, even low-level CMV replication that persists beyond the first year may contribute to fibrotic remodeling and late multiorgan failure, thereby resulting in higher 5-year mortality, even in the absence of EBV (Forker et al., 2019). In contrast, EBV is a B-cell tropic γ-herpesvirus whose lytic cycle is rapidly controlled by donor-derived EBV-specific CTLs once CD4^+^ T-helper cell function recovers (O’Mahony et al., 2009; Nikiforow et al., 2024). In the absence of profound and prolonged T-cell depletion, EBV DNAemia is usually an acute, self-limiting event that rarely produces direct end-organ toxicity. When CMV and EBV occur concurrently, the CMV-driven type I interferon response may accelerate EBV-specific CTL expansion via bystander activation, thereby shortening EBV persistence (Prockop et al., 2020; Toh et al., 2024). Thus, the inflammatory cytokine response that contributes to endothelial injury may also facilitate clearance of EBV-infected B cells. Therefore, long-term damage appears to be driven primarily by the CMV component. Consequently, patients with late isolated CMV reactivation may experience progressive vasculopathy, whereas those with transient dual viremia may achieve EBV clearance and remain at lower late-mortality risk, which may explain why CMV alone was associated with a stronger 5-year hazard than coreactivation in the survival model.

## 5 Limitations

This study has several limitations. The single-center retrospective design may introduce selection and surveillance biases, although standardized twice-weekly PCR monitoring and uniform preemptive algorithms were employed. Pharmacokinetic data for ganciclovir and rituximab were not available, precluding analysis of dose–efficacy relationships. Viral whole-genome sequencing was not performed; therefore, differential outcomes cannot be attributed to specific CMV or EBV strains, nor can the contribution of ultra-rare subclones that might escape T-cell recognition be determined. The study was conducted in a predominantly haploidentical, T-cell–replete setting with post-transplant cyclophosphamide; therefore, extrapolation to matched sibling or umbilical cord transplantation platforms should be interpreted with caution. The absence of donor CMV/EBV DNA measurements precluded risk stratification according to established donor/recipient (D/R) serostatus categories. Prospective studies incorporating complete donor virological data are warranted to validate these findings in the context of CMV-specific risk profiles. Finally, although the sample size was sufficient for multivariate modeling, it was underpowered to detect interactions within rarer subgroups, such as unrelated donor grafts or second transplant recipients. Despite these limitations, the consistency of the coreactivation signal across multiple sensitivity analyses, including landmark models excluding early deaths and competing risk regressions treating relapse and non-relapse mortality as distinct events, suggests that the findings are robust. Prospective, multicenter validation is warranted to confirm whether integrating dual viral load metrics into transplant algorithms may translate the survival benefit observed in this retrospective cohort into a reproducible causal improvement for all allo-HSCT recipients.

## 6 Conclusion

In conclusion, this single-center cohort of 202 patients demonstrates that CMV/EBV reactivation is not merely a by-product of allo-HSCT but a dynamic, time-dependent determinant of late mortality and relapse. These data support extending herpesvirus surveillance to 2 years post-transplantation, beyond the conventional 100-day monitoring period, and provide a framework for interventional trials targeting enhanced viral suppression, adoptive T-cell therapy, or thymic-enhancing agents. Until such strategies are validated, integrating serial CMV/EBV PCR monitoring into routine risk assessment may provide an accessible approach to refine prognosis, tailor immunosuppression, and potentially improve long-term survival after modern haploidentical transplantation.

## Data Availability

The raw data supporting the conclusions of this article will be made available by the authors, without undue reservation.
