# Supplementary material for: Late-phase impact of CMV/EBV reactivation on survival after hematopoietic stem-cell transplantation: a 5-year single-center cohort study
Source: Front Cell Infect Microbiol. 2026 Mar 25;16:1782595. doi: 10.3389/fcimb.2026.1782595 (PMC13058802; doi:10.3389/fcimb.2026.1782595)
Supplement: Supplementary file 1 [file Table1.docx]

| patients | groups | mortality cause |
| --- | --- | --- |
| 1 | Co-reactivation group | sepsis |
| 2 | Co-reactivation group | bad transplantation；respiratory failure |
| 3 | Co-reactivation group | relapse |
| 4 | Co-reactivation group | pulmonary infection；respiratory failure；GVHD |
| 5 | Co-reactivation group | multiple organ failure |
| 6 | Co-reactivation group | pulmonary infection |
| 7 | Co-reactivation group | relapse |
| 8 | Co-reactivation group | relapse |
| 9 | Co-reactivation group | sepsis； GVHD |
| 10 | CMV reactivation group | pulmonary infection；respiratory failure |
| 11 | CMV reactivation group | sepsis； liver failure |
| 12 | CMV reactivation group | pulmonary infection；respiratory failure |
| 13 | CMV reactivation group | pulmonary infection |
| 14 | CMV reactivation group | relapse |
| 15 | CMV reactivation group | pulmonary infection；respiratory failure；GVHD |
| 16 | CMV reactivation group | pulmonary infection；respiratory failure；GVHD |
| 17 | CMV reactivation group | pulmonary infection；respiratory failure |
| 18 | CMV reactivation group | sepsis |
| 19 | CMV reactivation group | relapse |
| 20 | CMV reactivation group | pulmonary infection；multiple organ failure |
| 21 | CMV reactivation group | pulmonary infection |
| 22 | CMV reactivation group | liver failure |
| 23 | CMV reactivation group | relapse |
| 24 | CMV reactivation group | bone marrow failure； epilepsy； coma |
| 25 | CMV reactivation group | relapse |
| 26 | CMV reactivation group | pulmonary infection；respiratory failure |
| 27 | CMV reactivation group | multiple organ failure |
| 28 | CMV reactivation group | pulmonary infection；respiratory failure；GVHD |
| 29 | CMV reactivation group | relapse |
| 30 | CMV reactivation group | relapse |
| 31 | No reactivation group | relapse |
| 32 | No reactivation group | pulmonary infection |
| 33 | No reactivation group | respiratory failure；serious myocarditis |

**Supplementary table S2.** **Causes of mortality**

Abbreviations: CMV, Cytomegalovirus; GVHD, graft-versus-host-disease
